# Erectile dysfunction among male patients receiving methadone maintenance treatment: focusing on anxiety-related symptoms

**DOI:** 10.1093/sexmed/qfae052

**Published:** 2024-08-23

**Authors:** Te-Chang Changchien, Tsung-Jen Hsieh, Yung-Chieh Yen

**Affiliations:** Department of Psychiatry, E-Da Hospital, Kaohsiung City 824, Taiwan; School of Medicine, College of Medicine, I-Shou University, Kaohsiung City 824, Taiwan; School of Medicine, College of Medicine, I-Shou University, Kaohsiung City 824, Taiwan; Department of Psychiatry, E-Da Hospital, Kaohsiung City 824, Taiwan; School of Medicine, College of Medicine, I-Shou University, Kaohsiung City 824, Taiwan

**Keywords:** methadone, erectile dysfunction, anxiety, alcohol

## Abstract

**Background:**

Erectile dysfunction (ED) in patients receiving methadone maintenance treatment (MMT) is a relatively neglected issue.

**Aim:**

In this study we sought to determine the actual prevalence of ED and risky sexual behaviors in patients receiving MMT and identify clinically relevant risk factors for ED, particularly mental health conditions, that may contribute to achieving holistic healthcare and improving treatment adherence in this patient population.

**Methods:**

A cross-sectional study of male Chinese MMT patients was conducted. Comprehensive demographic and clinical data regarding age, obesity, history of major mental and physical illness, HIV infection, other substance use, methadone dose/duration, and associated risky sexual behaviors were all collected. Assessment tools, including the 5-item International Index of Erectile Function, the Chinese Health Questionnaire, and the Taiwanese Depression Questionnaire were administered.

**Outcomes:**

The relationship between mental health–related factors and ED was fully analyzed and elaborated.

**Results:**

The prevalence of ED among male patients in a methadone maintenance therapy outpatient clinic was 55.7%. The prevalence rate of ED among the individuals treated for longer than 6 months was 56.8%, whereas that for untreated individuals was 52.0%. Additionally, methadone-treated individuals were older and had a higher proportion of condom use and drug-assisted sexual activity than untreated individuals. Pearson correlation revealed that higher Chinese Health Questionnaire and Taiwanese Depression Questionnaire scores were negatively correlated with lower scores on the 5-item International Index of Erectile Function. In the multivariate regression model, anxiety and other psychosomatic symptoms were associated with more severe ED, whereas individuals who consumed alcohol within the past month had less severe ED after adjustment for other demographic and clinical variables. The findings of the present study revealed no association between ED and methadone treatment duration or dosage.

**Clinical Implications:**

Healthcare professionals should discuss mental health issues in patients on MMT with ED, especially anxiety symptoms and recent alcohol use.

**Strengths and Limitations:**

This study is one of the few reports within the limited body of research highlighting a significant association of ED with anxiety-related symptoms in patients undergoing MMT. Our study had some limitations. First, the sample size of HIV-infected individuals was insufficient. Second, the cross-sectional study design could not definitively demonstrate a causal mechanism.

**Conclusion:**

In patients undergoing MMT, individuals who reported less severe anxiety symptoms and alcohol consumption in the past month tended to have less severe ED, regardless of the MMT duration or dosage.

## Introduction

Since the 1960s, methadone has been used as a detoxification and maintenance therapy for patients with heroin use disorder (HUD). Extensive clinical research has confirmed methadone maintenance therapy (MMT) to be safe and effective for opioid addiction treatment.^1^ This treatment allows individuals to take long-acting, well-absorbed, and legal methadone to reduce their dependence on heroin and avoid fatal and nonfatal opioid overdoses. Consequently, individuals with HUD can sustain a normal lifestyle, restore family and social relationships, and achieve harm reduction according to the harm-reduction principle, reducing the infectious complications of HUD and the transmission of blood-borne infections such as HIV and viral hepatitis through needle and diluent sharing by multiple users.^2^

According to the World Health Organization, sexual health refers to “a state of physical, emotional, mental, and social well-being related to sexuality.” Achieving sexual health depends on the extent to which sexual rights are respected, protected, and empowered. Patients with HUD are often marginalized in society and are stigmatized for being associated with antisocial behavior. Despite addiction being acknowledged as a brain disease and an important mental health issue, the human rights of individuals suffering from addiction remain suppressed and undervalued, especially those regarding sexual health and dysfunction, which are currently overlooked in clinical practice, particularly among individuals infected with HIV.

Since a trial of MMT began in Taiwan in 2006, HIV incidence among persons who inject drugs has shown a declining trend.^3^ However, in addition to the traditional emphasis on harm reduction, reduced medical costs, and social crime rates, the need to address psychiatric comorbidities within this population, such as depression, anxiety disorders, personality disorders, and suicide, is common and requires proactive management. Among these comorbidities, sexual dysfunction is a relatively neglected concern, particularly because it correlates with heroin, alcohol, and other substance addictions, depression, HIV infection, cardiovascular diseases, diabetes, and other risk factors.^4^ Thus, understanding the actual prevalence of sexual dysfunction in Chinese male patients undergoing MMT, identifying clinically relevant risk factors, and integrating these into clinical practice may lead to the identification of comprehensive healthcare objectives and potentially enhance treatment adherence. Similarly, studies have reported that sexual dysfunction can disrupt treatment compliance for depression, HIV, and hypertension. In contrast, identifying the risk factors for sexual dysfunction within this population could enhance the motivation for addiction cessation. Moreover, current reports indicate that the prevalence rate of sexual dysfunction among male patients undergoing methadone treatment ranges from 14% to 97%.^5-7^, with data on the Chinese population lacking. This variability suggests that many reported studies had limited sample sizes and lacked consistent and reliable measurement tools (questionnaires) to assess these phenomena. In the present study we aimed to investigate the prevalence of and factors associated with erectile dysfunction (ED) in male patients undergoing MMT, given that ED is one of the most common forms of sexual dysfunction^8^ and ED pathogenesis and risk factors have been well established (involving vascular, neurological, endocrine, and psychological factors), making them applicable to this specific population.^9^

Investigations and statistical analyses of resulting data have revealed that the transmission of HIV infection can be predominantly attributed to two major groups: men who have sex with men and injection drug users. Among injection drug users, the primary causes of HIV transmission are improper needle use (sharing or reuse) and risky sexual behavior. After compiling and reviewing 23 articles related to addiction treatment and HIV prevention strategies, Sorensen et al. found that methadone treatment provided clear and credible evidence for reducing HIV infections caused by needle use.^10^ However, evidence linking methadone to risky sexual behaviors is relatively limited.^11^ Therefore, the questions that we sought to answer the following questions in the present study: First, what are the differences in the rates of common demographic variables, alcohol and other drug use, clinical comorbidities, risky sexual behaviors, and HIV infection between the untreated HUD group compared to MMT group? Second, is there a significant correlation between ED and the duration and dosage of MMT, as well as mental health conditions such as depressive and anxiety symptoms in HUD patients ? Last, what are the factors associated with ED (especially mental health) among these Chinese patients?

## Methods

### Subjects

All participants were recruited from the methadone outpatient clinic of a general hospital in Kaohsiung City, Taiwan. The untreated group consisted of individuals diagnosed with HUD by psychiatrists upon initial admission to the psychiatric department. The methadone treatment group comprised individuals from the same outpatient clinic who had been undergoing MMT for at least 6 months. All of the participants were males. Before the interview, the respondents were required to complete a research questionnaire and provide informed consent indicating their willingness to participate. Data were collected using a self-administered questionnaire. Between January and June 2015, 106 research subjects were enrolled in our cross-sectional study, comprising 25 individuals in the untreated group and 81 in the MMT group. Individuals with no sexual experience within the past 6 months and those who had suffered from penile corporeal or neurological injury were excluded from the study. The Institutional Review Board of E-Da Hospital (Kaohsiung, Taiwan) approved the study design.

### Screening tools

The 5-item version of the International Index of Erectile Function (IIEF-5) was derived by Rosen et al. It is used to diagnose and grade ED, focusing on the assessment of ED and overall sexual satisfaction. It consists of 5 subitems evaluating confidence in achieving erection, maintenance of erection hardness before and after penetration, difficulty in completing intercourse, and satisfaction with sexual intercourse (scored from 1 to 5 for each item). Analysis of 1036 patients with ED and 116 control group members identified 21 points as the cutoff score for best discrimination (sensitivity = 0.98, specificity = 0.88). Subsequently, ED was classified as mild (score 17-21), mild to moderate (score 12-16), moderate (score 8-11), or severe (score 5-7).^12^ Regardless of the ED etiology, this scale has demonstrated excellent diagnostic quality.

The Taiwanese Depression Questionnaire (TDQ) is an 18-question, 0-3–point questionnaire used to screen for clinical depressive symptoms. The questionnaire is a culturally specific depression self-rating instrument used in Taiwan.^13^ The TDQ has a Cronbach’s α of 0.90 and sensitivity and specificity of 0.89 and 0.92, respectively. The cutoff score for the Taiwanese community population was ≥19 points.

The Chinese Health Questionnaire (CHQ) is a 12-question, 2–reverse question, 0-1–point questionnaire used to screen for somatic and psychic anxiety symptoms, social dysfunction, self-confidence, and hope, all suggestive of nonpsychotic and anxiety symptoms in common mental disorders (CMDs). The CHQ was derived from the General Health Questionnaire, with the addition of specially designed and culturally relevant items.^14^ In a community study, the questionnaire had a sensitivity and specificity of 70% and 95 %, respectively, for minor psychiatric morbidities.^15^ A Cronbach’s α of 0.84 and internal consistency of 0.79 were demonstrated for the CHQ.^16^ The cutoff for the Taiwanese community population was ≥5 points.

### Statistical analysis

Descriptive results regarding continuous variables are presented as the mean ± SD, and categorical variables are presented as numbers and percentages. First, a demographic comparison was conducted between the two groups of the study sample, focusing on the general individual and social characteristics of the untreated and MMT groups (such as sex, age, body mass index, occupation, educational level, and marital status), recent alcohol or other illicit drug use within the past month, clinical physical and psychiatric comorbidities (CHQ and TDQ scores), sexual risk behaviors in the last 6 months (including the number of sexual partners, condom use, engagement in sexual activity after substance use, and substance use of sexual partners), and ED (IIEF-5 scores). To validate multiple research hypotheses, we employed inferential statistics, including the chi-square, *t*-test, Pearson correlation, and linear regression analyses. We explored the differences between untreated and methadone-treated groups in terms of various demographic and clinical variables. Pearson correlation coefficients were computed to examine the relationship between ED (IIEF-5 scores) and methadone treatment variables (duration and dosage of methadone treatment) as well as depression and anxiety symptoms (CHQ and TDQ scores). Finally, the IIEF-5 scores were used as the dependent variable, and the above demographic variables, methadone treatment variables (duration and dosage of MMT), recent illicit drug or alcohol use within the past month, clinical comorbidities, sexual risk behavior within the last 6 months, and HIV infection were set as independent variables. Multivariate linear regression analysis was used to identify variables independently associated with ED. All analyses were performed using SPSS version 19.0 for Windows (IBM, United States).

## Results

### Demographic and clinical characteristics

**Figure 1:**
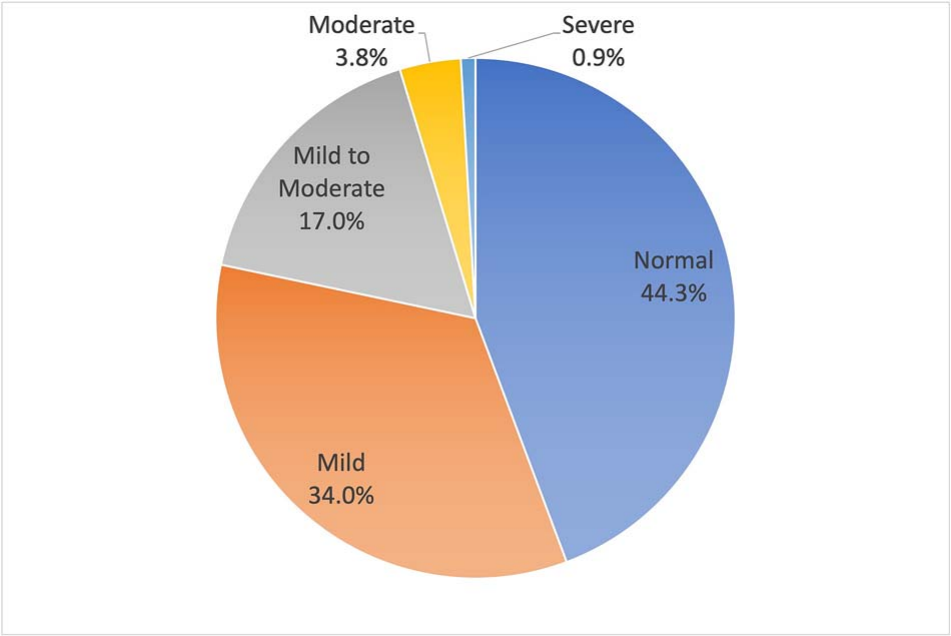
Prevalence and severity of ED among the 106 participants. ED, erectile dysfunction; IIEF-5, International Index of Erectile Function-5.

In terms of demographic characteristics, the average age of individuals undergoing methadone maintenance treatment was 43.15 ± 6.99 years, whereas that of untreated individuals was 39.20 ± 5.88 years. A *t*-test revealed a statistically significant difference between the two groups (*t* = −2.56; *P* = .012). Regarding risky sexual behavior, concerning condom use within the last 6 months, a significantly higher proportion of condom use was observed among those receiving treatment, accounting for 54.3%, compared to 28.0% among untreated individuals (chi-square = 6.63; *P* = .036). Additionally, among those receiving treatment, the proportion of drug-assisted sexual activity within the last 6 months was significantly lower (32.1%) than that in untreated individuals (56.0%) (chi-square = 4.65; *P* = .031). No significant differences were observed in the other demographic or clinical characteristics between the two groups (Table 1). In terms of the prevalence of ED, defined as the proportion of individuals with an IIEF-5 score of <21, the prevalence rate in the MMT clinic was 55.7%. The prevalence rate among those who had received treatment for longer than 6 months was 56.8%, whereas that for untreated individuals was 52.0%. The distribution of ED severity levels is shown in Figure 1: 47 (44.3%) individuals reported having normal erectile function, and 34.0%, 17.0%, 3.8%, and 0.9% of participants had mild, mild-to-moderate, moderate, and severe ED, respectively. When comparing scores based on IIEF-5, individuals undergoing methadone treatment had an average score of 19.83 ± 3.99, whereas untreated individuals had an average score of 19.68 ± 4.46. No statistically significant difference was observed between the two groups (*t* = −0.16; *P* = .876). In the context of the CHQ, respondents receiving methadone treatment had an average score of 3.72 ± 2.91, whereas the untreated individuals had an average score of 4.08 ± 3.21. No statistically significant difference was observed between the two groups (*t* = 0.53; *P* = .595). Similarly, in terms of scores on the TDQ, individuals undergoing methadone treatment had an average score of 10.72 ± 11.10, whereas the untreated individuals had an average score of 13.20 ± 12.50. No statistically significant difference was observed between the two groups (*t* = 0.95, *P* = .345) (Table 2).

**Table 1:** Demographics and clinical characteristics of the HUD patients.

	**Methadone treatment group (n = 81)**	**Untreated group (n = 25)**	***P*-value**
Age, y, mean ± SD	43.15 ± 6.99	39.20 ± 5.88	.012
Body mass index, mean ± SD	24.83 ± 4.02	23.95 ± 2.85	.314
Years of education, No. (%)			
0-9	34 (42.0)	15 (60.0)	.114
>9	47 (58.0)	10 (40.0)	
Joblessness, No. (%)	9 (11.1)	4 (16.0)	.5
Married, No. (%)	28 (34.6)	11 (44.0)	.393
Hypertension, No. (%)	10 (12.3)	1 (4.0)	.452
Diabetes, No. (%)	6 (7.4)	0 (0)	.332
HIV infection, No. (%)	10 (12.3)	3 (12.0)	1
Psychiatric history, No. (%)	24 (29.6)	7 (28.0)	.876
>1 sex partner, No. (%)	11 (13.6)	1 (4.0)	.286
Condom use, No. (%)	44 (54.3)	7 (28.0)	.036
Drug-assisted sex, No. (%)	26(32.1)	14(56.0)	.031
Partner with drug use, No. (%)	7 (8.6)	3 (12.0)	.697
Alcohol use within 1 mo, No. (%)	28 (34.6)	7 (28.0)	.542
Tobacco user, No. (%)	70 (86.4)	22 (88.0)	1
Amphetamine use history, No. (%)	40 (49.4)	16 (64.0)	.201

Abbreviation: HUD, heroin use disorder.

**Table 2:** Comparison of IIEF-5, CHQ, and TDQ scores between the untreated and methadone treatment groups.

	Methadone treatment group, mean ± SD (n = 81)	Untreated group, mean ± SD (n = 25)	*P*-value
IIEF-5 score	19.83 ± 3.99	19.68 ± 4.46	.876
CHQ score	3.72 ± 2.91	4.08 ± 3.21	.595
TDQ score	10.72 ± 11.10	13.20 ± 12.50	.345

Abbreviations: CHQ, Chinese Health Questionnaire; IIEF-5, International Index of Erectile Function-5; TDQ, Taiwanese Depression Questionnaire.

### Analysis of Pearson correlations

The IIEF-5 and CHQ scores showed a moderate negative correlation (*r* = −0.319; *P* = .001), and a low negative correlation was observed between IIEF-5 and TDQ scores (*r* = −0.262; *P* = .007). This indicates that as the severity of ED increases, mental health conditions, such as depression and anxiety, worsen. There was a weak positive correlation between MMT duration and treatment dosage (*r* = 0.241; *P* = .013); however, neither of these factors exhibited statistically significant correlations with ED or mental health conditions. A high positive correlation was observed between the CHQ and TDQ scores (*r* = 0.794; *P* < .001), suggesting a substantial mutual influence between the symptoms of depression and anxiety (Table 3).

**Table 3:** Pearson correlation depicting the associations among ED, MMT duration and dose, and mental health status.

	IIEF-5 score	MMT dose	MMT duration	CHQ score	TDQ score
IIEF-5 score	1				
MMT dose	0.036	1			
MMT duration	0.009	0.241^*^	1		
CHQ score	−0.319^**^	−0.094	−0.047	1	
TDQ score	−0.262^**^	−0.145	−0.087	0.794^**^	1

Abbreviations: CHQ, Chinese Health Questionnaire; IIEF-5, International Index of Erectile Function; MMT, methadone maintenance treatment; TDQ, Taiwanese Depression Questionnaire.

^*^ *P* < .05; **P < .01.

### Multivariate linear regression analysis

When considering the total score distribution of the IIEF-5 as the dependent variable in a linear regression model, the stepwise method yielded results indicating statistical significance in the final model for CHQ score and alcohol use within the past month as predictor variables for ED, after controlling for other demographic and clinical variables. Specifically, anxiety and other psychosomatic symptoms were associated with more severe ED (β = −.32; *P* = .001), whereas individuals who consumed alcohol within the past month had less severe ED (β = .19; *P* = .044) (only significant variables are shown in Table 4).

**Table 4:** Analysis of factors predicting ED in the multivariate linear regression model (No. = 106).^a^

	Unstandardized		β	*P*-value
	β	SE		
CHQ score	−.44	0.13	−.32	.001
Alcohol use within 1 mo	1.63	0.8	.19	.044

Abbreviations: CHQ, Chinese Health Questionnaire; ED, erectile dysfunction.

^a^Only variables with significant values are shown in the table.

## Discussion

Our main findings were that individuals undergoing methadone treatment were older than untreated individuals, with a higher proportion using condoms and a lower proportion engaging in drug-assisted sexual activity. Second, higher CHQ and TDQ scores were negatively correlated with lower IIEF-5 scores. Notably, worsening mental health conditions characterized by more severe anxiety symptoms are associated with more severe ED. Additionally, individuals who had consumed alcohol within the past month exhibited less severe ED.

The CHQ was used to assess the degree of somatic or psychic anxiety symptoms (predictive of CMDs) among patients with HUD. A meta-analysis of 16 studies revealed statistically significant associations between certain clinical factors and sexual dysfunction in patients undergoing MMT. In addition to mental disorders, these factors include age, hormonal analysis, and the dosage and duration of MMT.^17^ However, upon detailed examination of several related studies, it was observed that most of these studies focused primarily on investigating depressive symptoms, employing depression-focused scales with minimal emphasis on the impact of anxiety-related symptoms. The present study is one of the few reported studies within the limited body of research highlighting a significant association with anxiety-related symptoms. The CHQ, having undergone extensive clinical research in our country over the years and having gained international recognition with publications in top-tier journals, illustrates a potential doubling trend in the prevalence of probable CMDs in Taiwan, reaching 23.8%. This finding indicates that the mental health status of the Taiwanese population may have deteriorated proportionally over the past 2 decades owing to factors such as rising unemployment, divorce, and suicide rates.^18^ These trends align with the concept promoted by the World Health Organization in recent years: “No health without mental health.” The mental health of the Taiwanese population, especially vulnerable groups such as patients with HUD who are easily marginalized or stigmatized, should be prioritized, particularly considering its close association with the ED. Traditionally, the psychogenic aspects of ED have been associated with anxiety. The risk factors include male population of a younger age, lack of significant cardiovascular risk factors, and sudden and situational occurrence but with normal erectile function during masturbation or in the morning. The underlying causes may be related to performance anxiety (concerns about inadequate performance), relationship issues between partners, and individual traits (susceptibility to stress, tendency toward anxiety, and fear).^19^^,^^20^ In contrast, ED treatment has been demonstrated in a randomized, double-blind, placebo-controlled trial to alleviate mild to moderate depressive symptoms, restore self-esteem, and improve quality of life.^21^

Alcohol is a popular and readily accessible recreational substance. It is often used to alleviate performance anxiety, enhance sexual performance, and treat ED. The findings of this study revealed that patients who reported alcohol consumption in the past month exhibited milder ED. Similar studies support this notion, suggesting a hypothesis that attributes the etiology of ED to atherosclerosis in the arterial endothelium, sharing common pathogenic mechanisms with prevalent vascular diseases such as coronary artery disease and stroke.^22^ Previous evidence has demonstrated the protective effects of light to moderate alcohol consumption on cardiovascular diseases, prompting further investigations into whether similar results are observed in the ED context.^23^ A meta-analysis of 11 cross-sectional studies exploring the relationship between alcohol consumption and ED revealed a significant negative correlation between regular alcohol intake and ED.^24^ These findings suggest that consuming more than 8 standard drinks per week (where one standard drink equals 10 g of alcohol) significantly reduces the risk of ED. However, there were no significant findings for alcohol intake ranging from 1 to 7 units, indicating that the degree of alcohol consumption plays a crucial role, and moderate alcohol use is recommended for optimal protective effects. Another meta-analysis showed that light-to-moderate alcohol consumption correlated with a decreased risk of ED instead of high alcohol consumption, and further analysis revealed that the risk of ED increased quickly at very heavy alcohol consumption levels.^25^ Evidence has demonstrated that prolonged alcohol use can negatively affect sexual function and lead to ED. Studies on the effects of alcohol on physiological and endocrine functions have indicated that long-term alcohol use can result in decreased hormone secretion from the pituitary gland, testicular atrophy, and inhibition of testosterone production.^26^ Clinical studies have shown that patients with alcohol use disorder (previous alcohol dependence) assessed using the IIEF-15 exhibit a prevalence rate of ED of up to 74.7%.^27^ In summary, the possible reasons for the results of this study may be that the population meeting the criteria for the alcohol use questionnaire included individuals who may be low to moderate alcohol users who do not yet meet the diagnostic criteria for alcohol use disorder (which could worsen erectile function).

Regarding the link between illicit substances and sexual function, the public has the impression that the use of these drugs can increase sexual pleasure and performance. Similarly, substances could relieve anxiety, as the opioid system plays a crucial role in the neural modulation of anxiety through its modulatory effects on the GABAergic, serotonergic and glutamatergic systems.^28^ However, there are sufficient studies demonstrating the negative effects of illicit substances on sexual function. High doses of opiates (eg, heroin) have been shown to reduce blood testosterone levels, causing hypogonadism and sexual dysfunction.^29^ Another possible mechanism for sexual dysfunction involves brain circuitry and neurotransmission, with opiates abnormally activating and conditioning the mesolimbic dopaminergic reward circuitry. This mechanism is a major source of reward and behavioral stimulation associated with primary rewards such as food and sexual behavior, and opiates act as a powerful positive reinforcer that keeps addicts engaged in repeated drug-seeking behavior.^30^ Based on current evidence, the relationship between dosage (or duration) of opiate use and testosterone concentrations or ED remains controversial and inconclusive. In addition, the results of our study also showed no association between ED and methadone treatment duration or dosage.

An effective harm reduction strategy, MMT has been proven to minimize negative health, social and legal impacts associated with heroin use. Our study also supported that individuals undergoing methadone treatment had higher rates of condom use and lower rates of participation in drug-assisted sexual activity than untreated individuals. A recent review article proved the importance of harm reduction and prevention strategies for sexual health, particularly the evidence from illicit drugs and alcohol.^31^ Furthermore, the CHQ and alcohol use were the only two factors in this study that showed a significant association with ED, a finding that raises two questions: are individuals with more severe anxiety symptoms more likely to engage in alcohol use (as a coping mechanism for anxiety or discomfort) and sexual risk behaviors, further affecting erectile function? Or does alcohol lead to loss of control which may expose individuals to sexual risk behaviors, which may increase anxiety and thus ultimately affect erectile function? The possibility of an interaction between these two factors should be explored in future studies for validation purposes.

Our study had some limitations. First, it should be noted that the sample size was relatively small. Cultural factors can have an impact on sexual health, stigma toward persons who inject drugs (especially with HIV infection), and availability of treatment. In addition, patients with HUD usually have limited insight, coupled with lower-middle socioeconomic status, making it more difficult to persuade them to participate in research programs, let alone explore the private issue of sexual dysfunction. The sample size of this study is influenced by both of these possible factors. Small sample size is a limitation that affects the generalizability of research results. So, post hoc power analysis was performed to evaluate the statistical power of significant results at a significance level of.05. The post -hoc power calculation was conducted using the G^*^Power analysis program.^32^ These post hoc power analyses have confirmed that our methods have adequate power for detecting significant results. Furthermore, should the number of HIV-infected individuals enrolled reach an adequate level, it is anticipated that a stratified analysis will be conducted to identify factors predicting ED specifically within the HIV-infected subgroup. Second, the cross-sectional study design did not enable a definitive demonstration of a causal mechanism or predictive power. Therefore, confirming the preliminary findings of this study would enhance the value of future research, which could be conducted in the form of a prospective study that incorporates blood sex hormone tests and more detailed study tools (such as refining the categorization of alcohol use levels and even the severity of the alcohol dependence screening questionnaire).

## Conclusion

The findings of the present study revealed no association between ED and MMT duration or dosage. However, mental health status may also play a role, as more severe anxiety symptoms are associated with worsening ED. Alcohol use (in dealing with ED) may also present a two-fold challenge. Theses conclusions can be used in patient education, highlighting the importance of considering psychiatric treatment if there is a need to improve the ED.
